# Nachweis der *BRAF*-V600E-Mutation beim metastasierten kolorektalen Karzinom: Ein QuIP-Ringversuch

**DOI:** 10.1007/s00292-021-01022-8

**Published:** 2021-11-22

**Authors:** Korinna Jöhrens, Josephine Fischer, Markus Möbs, Klaus Junker, Jutta Kirfel, Sven Perner, Silke Laßmann, Martin Werner, Vanessa Borgmann, Hendrik Bläker, Michael Hummel

**Affiliations:** 1 Institut für Pathologie, Universitätsklinikum Carl Gustav Carus, TU Dresden, Dresden, Deutschland; 2 Qualitätssicherungs-Initiative Pathologie QuIP GmbH, Berlin, Deutschland; 3 Institut für Pathologie, Campus Charité Mitte, Charité – Universitätsmedizin Berlin, Virchowweg 15, 10117 Berlin, Deutschland; 4 Zentrum für Pathologie, Klinikum Bremen-Mitte, Bremen, Deutschland; 5 Universitätsklinikum Schleswig-Holstein, Campus Lübeck, Lübeck, Deutschland; 6 Institut für Klinische Pathologie, Universitätsklinikum Freiburg, Freiburg, Deutschland; 7 Institut für Pathologie und Neuropathologie, Universitätsklinikum Tübingen, Tübingen, Deutschland; 8 Universitätsklinikum Leipzig, Leipzig, Deutschland

**Keywords:** Kolorektales Karzinom, Molekularpathologie, BRAF Mutation, Ringversuche, Präzisionsonkologie, Cororectal cancer, Molecular pathology, BRAF mutation, Round robin test, Precision oncology

## Abstract

Ringversuche sind ein wichtiges Instrument zur Qualitätssicherung. Dies betrifft in zunehmendem Maße auch die molekulare Diagnostik in der Pathologie, von deren Ergebnissen Therapieentscheidungen in der Präzisionsonkologie direkt abhängen. Beim metastasierten kolorektalen Karzinom (mKRK) stand bisher der Nachweis von *KRAS*-und *NRAS*-Mutationen im Vordergrund, deren Abwesenheit eine Therapie mit EGFR-blockierenden Antikörpern ermöglicht. Nun ist BRAF als weiterer prädiktiver Marker hinzugekommen, da mKRK Patienten mit einer *BRAF*-V600E-Mutation nach systemischer Vortherapie von einer Behandlung mit Encorafenib (einem BRAF-Inhibitor) in Kombination mit Cetuximab (Anti-EGFR-Antikörper) profitieren. Aufgrund der 2020 erfolgten Zulassung für diese Behandlung ist es wichtig, dass der diagnostische Nachweis einer *BRAF*-V600E-Mutation zuverlässig in den Pathologien durchgeführt werden kann. Daher wurde dieser Ringversuch durchgeführt, bei dem der Nachweis der *BRAF*-V600E-Mutation entweder mittels Immunhistochemie oder molekularer Verfahren erfolgen konnte. Die Ergebnisse des Ringversuchs belegen eindeutig, dass derzeit die molekulare *BRAF*-V600E-Bestimmung dem immunhistologischen Nachweis überlegen ist.

## Abstract (en)

Round robin testing is an important instrument for quality assurance. Increasingly, this also applies to the results of molecular diagnostics in pathology, which directly influence therapy decisions in precision oncology. In metastatic colorectal carcinoma (mCRC), the focus has been on detecting *KRAS* and *NRAS* mutations, whose absence allows therapy with EGFR blocking antibodies. Recently, BRAF has been added as another predictive marker, since mCRC patients with *BRAF* V600E mutation benefit significantly from treatment with encorafenib (a BRAF inhibitor) in combination with cetuximab (anti-EGFR antibody) after systemic therapy. Due to the approval of this treatment in 2020, it is a pre-requisite that *BRAF* V600E mutation detection in diagnostic pathologies is reliably performed. Therefore, this round robin test with *BRAF* V600E testing either by immunohistochemistry or molecular methods was performed. The round robin test results demonstrate that molecular BRAF V600E detection is currently clearly superior to immunohistochemical detection.

Das Kolorektalkarzinom ist eine der häufigsten bösartigen Tumorerkrankungen. Seit Juni 2020 steht mit der EMA-Zulassung von Encorafenib (BRAF-Inhibitor) in Kombination mit dem EGFR-blockierenden Antikörper Cetuximab erstmals eine chemotherapiefreie gezielte Therapieoption beim *BRAF*-V600E-mutierten metastasierten kolorektalen Karzinom zur Verfügung. Um die Qualität und Reproduzierbarkeit der *BRAF*-V600E-Testung in den pathologischen Einrichtungen zu überprüfen, wurde von der QuIP GmbH ein Ringversuch organisiert.

Das kolorektale Karzinom ist bei Frauen nach dem Mammakarzinom die zweithäufigste bösartige Tumorerkrankung, bei Männern die dritthäufigste nach Prostata- und Lungenkrebs und umfasst insgesamt fast 60.000 Neuerkrankungen pro Jahr in Deutschland. Etwa 23.000 Darmkrebspatienten versterben jährlich an ihrer Erkrankung und das relative 5‑Jahres-Überleben liegt bei Männern und Frauen bei etwa 60 % ([9], Tab. 3.6.1) [11]. Die zielgerichtete biomarkergetriebene Therapie hat beim Kolorektalkarzinom schon vor über 10 Jahren mit dem Nachweis von *KRAS*-Mutationen begonnen und nimmt durch weitere Biomarker immer weiter an Bedeutung zu. Im Falle der Abwesenheit von *RAS*-Mutationen besteht die Möglichkeit mit der Blockade von EGFR, eine zielgerichtete Therapieoption verwenden zu können. Es ist auch schon längere Zeit bekannt, dass 8–12 % der Kolorektalkarzinome mit einer* BRAF*-Mutation vergesellschaftet sind [3].

BRAF spielt eine wichtige Rolle im MAPK*-*Signalweg und ist damit an der Kontrolle und Steuerung des Zellwachstums entscheidend beteiligt. Während dieser Prozess unter physiologischen Bedingungen sehr engmaschig kontrolliert abläuft, führen bestimmte *BRAF*-Veränderungen – wie die V600E-Mutation – zu einer konstitutiven Aktivierung der Kinaseaktivität, die nicht mehr der physiologischen Kontrolle unterliegt. Damit kann ein unkontrolliertes Zellwachstum entstehen und zur Pathogenese des metastasierten kolorektalen Karzinom (mKRK) beitragen [8]. Der weitaus größte Teil der *BRAF-*Mutationen finden sich beim mKRK an der Aminosäureposition 600 und bestehen im Austausch von Valin (V) durch Glutaminsäure (E). *BRAF*-Mutationen an anderen Positionen oder Ersatz des V600 durch eine andere Aminosäure finden beim mKRK nur sehr selten statt und haben in diesem Kontext nur untergeordnete Bedeutung [7, 12]. Das *BRAF*-mutierte mKRK selbst weist eine molekulare Heterogenität auf [2, 4]. Diese Heterogenität spiegelt sich auch in den 4 „consensus molecular subtypes“ (CMS) wider. *BRAF*-Mutationen finden sich zwar überwiegend in der Gruppe CMS1 („MSI immune“, 42 %), treten aber auch in CMS2 („canonical“, 1 %), CMS3 („metabolic“, 16 %) und CMS4 (mesenchymal, 7 %) auf. Es treten nur äußerst selten primäre *BRAF*-Mutationen zusammen mit *KRAS*-Mutationen auf [12].

Bei Vorliegen eines mikrosatelliteninstabilen mKRK zusammen mit einer *BRAF-*V600E-Mutation kann in der Erstlinie eine immunonkologische Therapie mit Pembrolizumab durchgeführt werden, die im Rezidivfall von einer Therapie mit Encorafenib und Cetuximab abgelöst werden kann (Fachinformation Keytruda® 2021, Fachinformation Braftovi® 2021). Jedoch existieren zum aktuellen Zeitpunkt noch keinerlei klinische Studien zur besten Therapieabfolge bezüglich dieser beiden Behandlungsprinzipien. Bei Patienten mit mikrosatellitenstabilem mKRK und einer *BRAF*-V600E-Mutation kommen gemäß Leitlinienempfehlung die kombinierte Behandlung aus klassischer Chemotherapie und zielgerichteter Behandlung in Betracht [1, 12]. Unabhängig vom Mikrosatellitenstatus kann die chemotherapiefreie Kombination aus Encorafenib und Cetuximab beim *BRAF*-V600E-mutierten mKRK generell nach systemischer Vortherapie (unabhängig davon, ob diese in adjuvanter oder palliativer Intention gegeben wurde) eingesetzt werden [6].

Seit Juni 2020 steht mit der EMA-Zulassung von Encorafenib in Kombination mit dem EGFR-blockierenden Antikörper Cetuximab auf der Basis der Studie BEACON CRC [5] erstmals eine chemotherapiefreie gezielte Therapieoption zur Verfügung. Durch die Zulassung der *BRAF*-V600E-Mutation als therapeutischen Marker beim mKRK findet diese Konstellation Eingang in das (molekular-)diagnostische Portfolio vieler Institute für Pathologie. Erfreulicherweise kann für den molekularen bzw. immunhistochemischen Nachweis der *BRAF*-V600E-Mutation auf zumeist umfangreiche Vorerfahrung bei malignen Melanomen zurückgegriffen werden [10].

## Methodik

### Ringversuchsaufbau

Um die Qualität und Reproduzierbarkeit in den pathologischen Einrichtungen zu überprüfen, wurde von der QuIP GmbH ein Ringversuch organisiert, der den Nachweis der *BRAF-*V600E-Mutation auf Proteinebene mittels Immunhistochemie (IHC) mit einem Antikörper gegen V600E-mutiertes BRAF und zum Nachweis auf DNA-Ebene verschiedene molekulare Verfahren vorgesehen umfasste. Der hier beschriebene Ringversuch hat sich aufgrund der Ergebnisse klinischer Studien sowie der Vorgaben aus der Zulassung der EMA auf den Nachweis der *BRAF*-V600E-Mutation begrenzt.

### Interner Ringversuch

Das Lead-Institut (Charité Berlin) suchte aus ihrem Archiv insgesamt 15 Fälle von Kolorektalkarzinomen mit und ohne *BRAF*-V600E-Mutation heraus. Um die Eignung dieser Fälle für einen Ringversuch zu überprüfen, wurde ein interner Ringversuch, also eine dem offenen Ringversuch vorgeschaltete Testung des Probenmaterials durchgeführt. Die Organisation des internen Ringversuchs übernahm das Institut für Pathologie der Charité Berlin. Die erste Überprüfung der 15 ausgewählten Fälle übernahmen die Charité Berlin mittels molekularer Untersuchungen (Next Generation Sequencing, NGS) und das kooperierende Lead-Institut (Universitätsklinikum Dresden) mittels IHC. Die Gegentestung mit molekularen Methoden wurde vom Klinikum Bremen-Mitte, dem Universitätsklinikum Schleswig-Holstein, Campus Lübeck, dem Universitätsklinikum Freiburg und dem Universitätsklinikum Tübingen übernommen. Hierfür wurden den 4 Panelinstituten je Fall 3 Leerschnitte á 4 µm zur Verfügung gestellt. Die Ergebnisse aus den molekularen Methoden waren aus allen 5 Instituten (Lead- und Panelinstitute) bei 14 Fällen konkordant, lediglich ein Fall konnte von einem Institut nicht ausgewertet werden. Die Gegentestung für den IHC-Teil übernahmen das Universitätsklinikum Freiburg und das Universitätsklinikum Leipzig. Den beiden Panelinstituten wurden je Fall 2 Leerschnitte á 4 µm zur Verfügung gestellt. Bei der Auswertung der immunhistochemischen Färbungen wurden 11 Fälle gleich bewertet und 4 Fälle als Grenzfälle identifiziert. Als mögliche Ursache werden die unterschiedlichen Färbeprotokolle vermutet. Da die gleichen Fälle in der molekularen Untersuchung keine diskrepanten Ergebnisse zeigten und diese Fälle aus dem diagnostischen Alltag stammen, wurde ein heterogen bewerteter Fall in den offenen Ringversuch eingeschlossen. Die 10 für der Ringversuch ausgewählten Fälle wiesen einen Tumorzellgehalt von 50–90 % auf und waren 10–133 Monate alt (Tab. 1).

**Table 1:** 

Fall-Nr.		Mutationsstatus	Alter^a^ des Blocks (in Monaten)	Tumorzellgehalt (in %)	
MolPath	IHC			Erster Schnitt	Letzter Schnitt
1	2	V600E	133	80	70
2	5	V600E	133	60	70
3	4	WT	26	70	50
4	7	V600E	31	90	80
5	6	WT	24	70	90
6	1	V600E	26	70	90
7	8	V600E	10	80	80
8	10	WT	24	80	75
9	3	V600E	20	70	80
10	9	V600E	20	80	70

*IHC* Immunhistochemie

^a^Das Alter des Blocks wurde berechnet als die Spanne zwischen der Erstbefundung des Blocks bis zum Beginn des Ringversuchs am 02.06.2020

### Offener Ringversuch

Die Teilnehmer erhielten 10 Fälle mit jeweils 2 Schnitte á 4 µm für den immunhistochemischen Ringversuch und 3 Schnitte á 4 µm für den molekularen Ringversuch. Die Teilnehmer hatten 28 Werktage Zeit, um die Analysen durchzuführen und die Ergebnisse online an die QuIP zurückzumelden. Für jeden richtig bewerteten Fall wurden 2 Punkte vergeben, sodass eine maximale Punktzahl von 20 erreicht werden konnte. Sofern die Ergebnisermittlung aufgrund technischer Probleme nicht möglich war, wurde ein Punkt für diesen Fall vergeben (diese Option war nur für einen Fall möglich). Für eine erfolgreiche Teilnahme mussten mindestens 18 Punkte (90 %) erreicht werden. Von einer Teilnahme kann nur ausgegangen werden, wenn die pathologischen Einrichtungen Ihre Ergebnisse fristgerecht eingereicht haben. Insgesamt haben 51 Institute an diesem Ringversuch teilgenommen, wovon 42 Teilnehmer die *BRAF*-V600E-Mutationsuntersuchung mittels molekularer Verfahren und 9 Teilnehmer die IHC durchführten.

## Ergebnisse

### *BRAF*-V600E-Mutationsnachweis mittels molekularer Verfahren

Von insgesamt 42 Teilnehmern, die ein Ergebnis eingereicht haben, konnte allen 42 Teilnehmern (100 %) eine erfolgreiche Teilnahme bescheinigt werden. Es gab keine Häufungen von „falschen“ Bewertungen der 10 Ringversuchsfälle (Tab. 2).

**Table 2:** 

Fall	1	2	3	4	5	6	7	8	9	10
Positiv	*42*	*42*	1	*42*	0	*42*	*42*	0	*42*	*42*
Negativ	0	0	*40*	0	*42*	0	0	*42*	0	0
Nicht auswertbar	0	0	1	0	0	0	0	0	0	0

Von 420 ausgeführten Einzelanalysen waren 418 (99,5 %) korrekt, ein Ergebnis (0,2 %) war falsch positiv und eine Probe (0,2 %) war technisch nicht auswertbar (Tab. 3). Die aus dem Ringversuch abgeleitete Sensitivität für die molekulare Analyse liegt damit bei 100,0 % (294/294), die Spezifität bei 99,2 % (124/125).

**Table 3:** 

Anzahl der Teilnehmer	Richtig positiv	Richtig negativ	Falsch positiv	Falsch negativ	Nicht auswertbar	Punkte	Erfolg
40	7	3	0	0	0	20	Ja
1	7	2	0	0	1	19	Ja
1	7	2	1	0	0	18	Ja

Die für den molekularen Ringversuch genutzten Methoden waren in 2 Hauptkategorien unterteilt (Tab. 4): 1) kommerzielles All-In-One-Testverfahren (11/42 Teilnehmern, 26,2 %), welches vollautomatisiert Gewebeschnitte bis hin zum Ergebnis analysiert (Idylla System), und 2) labortechnische Einzelprozessierung der DNA-Extraktion gefolgt von weiteren molekularen Verfahren.

**Table 4:** 

Kategorie	Hersteller	Verwendetes DNA-Extraktionskit	Teilnehmer	Mit Erfolg (%)
Vollautomatisiert	Biocartis, Mecheln, Belgien	Idylla™ BRAF Mutation Assay	11	11 (100)
Labortechnische Einzelprozessierung	Analytik Jena GmbH, Jena, Deutschland	innuPREP FFPE DNA Kit-IPC16	1	1 (100)
	Promega GmbH, Waldorf, Deutschland	Maxwell 16 FFPE (Plus) LEV DNA Purification Kit	5	5 (100)
	Promega	Maxwell 16 LEV Blood DNA Kit	1	1 (100)
	Promega	Maxwell 16 LEV RNA FFPE Purification Kit	2	2 (100)
	Promega	Maxwell RSC DNA FFPE Kit	4	4 (100)
	Promega GmbH	ReliaPrep™ FFPE gDNA Miniprep System	1	1 (100)
	Qiagen GmbH, Hilden, Deutschland	QIAamp DNA FFPE Tissue Kit	4	4 (100)
	Qiagen	QIAamp DNA Micro Kit	3	3 (100)
	Qiagen	QIAsymphony DSP DNA Mini Kit	1	1 (100)
	Qiagen	RNeasy FFPE Kit	2	2 (100)
	STRATIFYER Molecular Pathology GmbH, Köln, Deutschland	XTRAKT FFPE Kit	1	1 (100)
	Zymo Research, Irvine, USA	Quick-DNA™ FFPE Kit	1	1 (100)
	–	Andere	5	5 (100)

**Figure 1:**
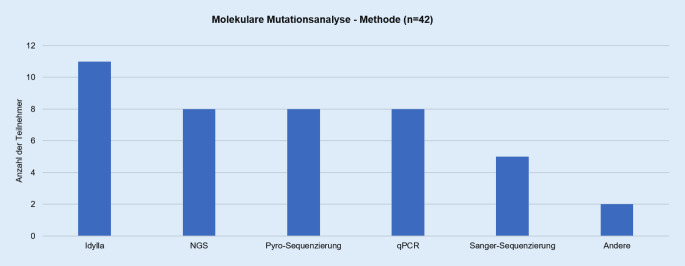

Die nach DNA-Extraktion verwendeten Methoden zum Nachweis einer *BRAF-*V600E-Mutation (Abb. 1) schlossen Parallelsequenzierung (NGS), Pyrosequenzierung, qPCR(quantitative Polymerase-Kettenreaktion)-Verfahren und Sangersequenzierung mit ein (Tab. 5).

**Table 5:** 

Kategorie	Methode	Hersteller	Plattform	Teilnehmer	Mit Erfolg (%)
Vollautomatisiert	–	Biocartis, Mecheln, Belgien	Idylla	11	11 (100)
Labortechnische Einzelprozessierung	*NGS*	Illumina, Berlin, Deutschland	Miniseq	1	1 (100)
		Illumina	MiSeq	2	2 (100)
		Illumina	NextSeq	1	1 (100)
		Thermo Fisher Scientific, Dreiach, Deutschland	Ion GeneStudio S5	3	3 (100)
		Thermo Fisher	Ion Personal Genome Machine™ (PGM™)	1	1 (100)
	*Pyrosequenzierung*	Qiagen, Venlo, Niederlande	PyroMark	8	8 (100)
	*qPCR*	Agilent Technologies, Santa Clara, USA	Stratagene Mx3005P	1	1 (100)
		Roche Diagnostics GmbH, Grenzach, Deutschland	LightCycler® 480	1	1 (100)
		Thermo Fisher Scientfic, Dreiach, Deutschland	7500 Real-Time PCR System	1	1 (100)
		Thermo Fisher	Arktik Thermal Cycler	1	1 (100)
		Thermo Fisher	Quant Studio	2	2 (100)
		Zytomed Systems GmbH, Berlin, Deutschland	SLAN-96S	1	1 (100)
		–	Andere	1	1 (100)
	*Sanger-Sequenzierung*	Thermo Fisher	Quant Studio	1	1 (100)
		Applied Biosystems, Waltham, USA	Genetic Analyzer 3500	1	1 (100)
		–	Andere	3	3 (100)
	*HRMA, Sanger-Seq. & allelspez. PCR*	Roche	LightCycler® 480	1	1 (100)
	*Hybridisierung*	–	Thermocycler und Wasserbad	1	1 (100)

Die Teilnehmer nutzen unterschiedliche Kits bzw. Panels sowie „laboratory developed tests“ (LDT) für den Nachweis der *BRAF-*V600E-Mutation (Tab. 6).

**Table 6:** 

Kategorie	Methode	Hersteller	Plattform	Teilnehmer
Vollautomatisiert	–	Biocartis, Mecheln, Belgien	Idylla™ BRAF Mutation Assay	11
Labortechnische Einzelprozessierung	*NGS*	Agilent Technologies, Santa Clara, USA	Tumor Hotspot Panel	1
		Qiagen GmbH, Hilden, Deutschland	QIAseq Human Actionable Solid Tumor Panel	1
		Qiagen	QIAseq Targeted DNA Custom Panel	1
		Thermo Fisher Scientific, Dreiach, Deutschland	Ion AmpliSeq™ Cancer Hotspot Panel v2	1
		Thermo Fisher	Ion AmpliSeq™ Colon and Lung Cancer v2	1
		Thermo Fisher	Oncomine Focus Assay	2
		–	Amplikon BRAF Exon 15	1
	*Pyrosequenzierung*	Qiagen	Therascreen BRAF Pyro Kit	4
		–	Laboratory Developed Tests (LDT)	4
	*qPCR*	Amoy Diagnostics, Xiamen, China	BRAF V600 Mutations Detection Kit	1
		EntroGen, Woodland Hills, USA	BRAF Mutation Analysis Kit II for Real-Time PCR	1
		Roche Diagnostics GmbH, Grenzach, Deutschland	BRAF/NRAS Mutation Test (LSR)	1
		Thermo Fisher	BRAF TaqMan® Mutation Detection Assays	1
		ViennaLab Diagnostics GmbH, Wien, Österreich	BRAF 600/601 StripAssay®	1
		–	Laboratory Developed Tests (LDT)	2
		–	Andere	1
	*Sanger-Sequenzierung*	SCIEX, Framingham, USA	DTCS QUICK START KIT	1
		–	Laboratory Developed Tests (LDT)	2
		–	Andere	2
	*HRMA, Sanger-Seq. & allelspez. PCR*	–	Laboratory Developed Tests (LDT)	1
	*Hybridisierung*	Medipro (ViennaLab Diagnostics GmbH)	BRAF KIT	1

### *BRAF*-V600E-Mutationsnachweis mittels IHC

Von insgesamt 9 Teilnehmern, die ein Ergebnis eingereicht haben, konnte für 6 Teilnehmer (67 %) eine erfolgreiche Teilnahme bescheinigt werden. Insbesondere bei den Fällen 1 und 5 (Tab. 7) gab es von den Referenzwerten abweichende Beurteilungen. Zur weiteren Klärung der diskrepanten Ergebnisse (technische Ebene/Färbung oder Interpretation) wurden alle Testsets mit den durch die Teilnehmer durchgeführten immunhistochemischen Färbungen zur Nachbegutachtung an das kooperierende Lead-Institut UK Dresden gesendet. Bei dem Fall 5 handelte es sich um den schwierigen Fall, der bereits im internen Ringversuch heterogen bewertet wurde. Aufgrund der interpretatorischen Schwierigkeiten wurde dieser Fall aus der Wertung ausgeschlossen, d. h. alle Teilnehmer bekamen hierfür die volle Punktzahl. Der Fall 1 blieb in der Wertung und war auch im Review eindeutig positiv. Zumeist war eine schwache Färbeintensität die Ursache für die Fehlbewertung durch die Teilnehmer.

**Table 7:** 

Fall	1	2	3	4	5	6	7	8	9	10
Positiv	*5*	*9*	*9*	2	*3*	0	*7*	*9*	*9*	0
Negativ	4	0	0	*7*	*6*	*9*	1	0	0	*9*
Nicht auswertbar	0	0	0	0	0	0	1	0	0	0

Von 90 ausgeführten Einzelanalysen waren 73 (81,1 %) korrekt, 7 Einzelanalysen (7,8 %) waren falsch und 10 (11,1 %) waren technisch nicht auswertbar (Tab. 8). Die Angaben zu Fall 5 zählen hierbei nicht als falsche Einzelanalysen, sondern wurden als nicht auswertbar gezählt. Die sich daraus ergebene Sensitivität für die IHC liegt bei 90,6 % (48/53), die Spezifität bei 92,6 % (25/27).

**Table 8:** 

Anzahl der Teilnehmer	Richtig positiv	Richtig negativ	Falsch positiv	Falsch negativ	Nicht auswertbar	Punkte	Erfolg
4	6	3	0	0	1	20	Ja
1	5	3	0	1	1	18	Ja
1	6	2	1	0	1	18	Ja
1	5	2	1	1	1	16	Nein
1	4	3	0	2	1	16	Nein
1	4	3	0	1	2	18	Nein

**Figure 2:**
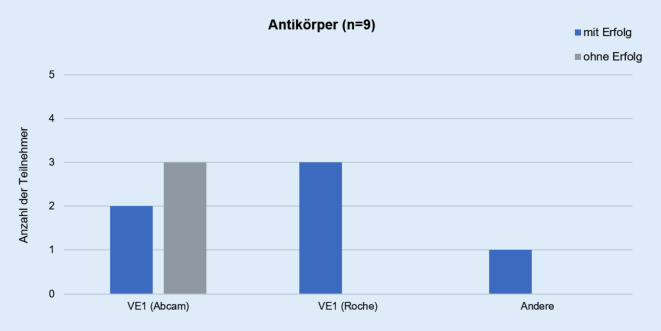

Für die Detektion der Proteinexpression des V600E-mutierten BRAFs verwendeten die Mehrzahl der Teilnehmer den Klon VE1 von Abcam (Cambridge, UK; 56 %) oder Roche Roche Diagnostics GmbH (Grenzach, Deutschland; 33 %). Die Inkubationszeiten variierten stark und lagen zwischen 9 und 92 min (Tab. 9). Auffallend ist, dass der Einsatz eines LTDs mit dem Klon VE1 von Abcam mit einer Verdünnung von 1:100 zu keiner erfolgreichen Teilnahme führte (Tab. 9, Abb. 2).

**Table 9:** 

Teilnehmer	Erfolg	Antikörper	Inkubationszeit in min	Verdünnung
1	Ohne Erfolg	VE1 (Abcam, Cambridge, UK)	92	1:100 (10 µg/ml)
2	Ohne Erfolg	VE1 (Abcam)	60	1:100 (10 µg/ml)
3	Ohne Erfolg	VE1 (Abcam)	8	1:100 (10 µg/ml)
4	Mit Erfolg	VE1 (Abcam)	15	1:50 (20 µg/ml)
5	Mit Erfolg	VE1 (Abcam)	20	1:50 (20 µg/ml)
6	Mit Erfolg	VE1 (Roche Diagnostics GmbH, Grenzach, Deutschland)	32	Ready-to-use (12 µg/ml)
7	Mit Erfolg	VE1 (Roche)	32	Ready-to-use (12 µg/ml)
8	Mit Erfolg	VE1 (Roche)	16	Ready-to-use (12 µg/ml)
9	Mit Erfolg	Andere	15	1:100 (10 µg/ml)

**Figure 3:**
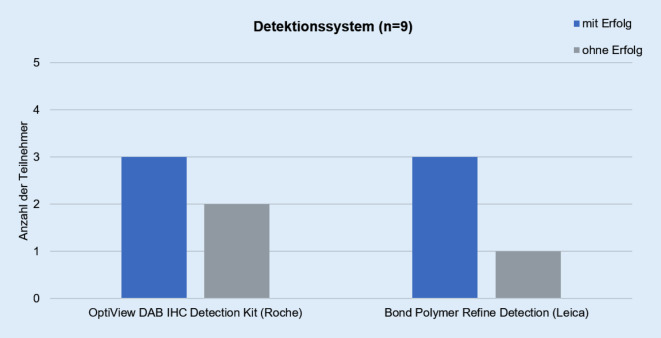

**Figure 4:**
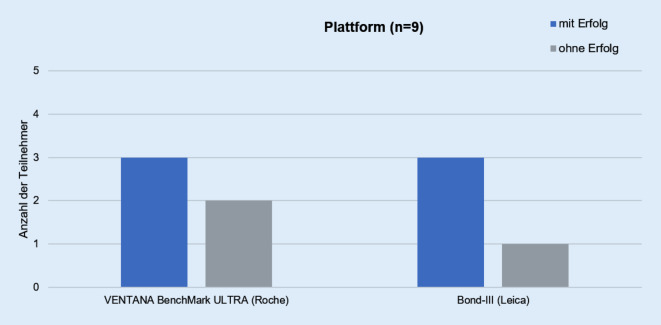

Die Teilnehmer verwendeten für die Detektion der V600E-mutierten BRAF-Proteinexpression das OptiView DAB IHC Detection Kit von Roche (56 %) oder das Bond Polymer Refine Detection Kit von Leica (Wetzlar, Deutschland; 44 %; Abb. 3). Die teilnehmenden Institute färbten die Testschnitte am häufigsten mit dem VENTANA BenchMark ULTRA (5 Teilnehmer, 56 %), gefolgt von dem Bond-III von Leica (4 Teilnehmer, 44 %; Abb. 4).

## Diskussion

Zusammenfassend kann festgestellt werden, dass die überwiegende Teilnehmerzahl (42/51) sich für den molekularen Nachweis entschied und lediglich 9 Institute die Immunhistochemie durchgeführt haben. Die Teilnehmer des molekularen Ringversuchsteils für den *BRAF*-V600E-Mutationsnachweis haben trotz der großen Heterogenität der eingesetzten Verfahren (von vollautomatisierter Analyse [DNA + qPCR, „All-in-One“], DNA-Extraktion mit qPCR bis hin zu DNA-Extraktion mit PCRs und anschließender Sequenziermethoden) alle den Ringversuch erfolgreich durchlaufen. Im Gegensatz hierzu haben nur zwei Drittel der Teilnehmer, die Immunhistochemie zum Nachweis der *BRAF*-V600E-Mutation gewählt haben, den Ringversuch erfolgreich durchlaufen, obwohl ein Fall mit anspruchsvoller Interpretation nicht in die Bewertung eingeflossen ist. Ursache für die nicht erfolgreiche Teilnahme erscheint mehrheitlich eine Bewertung von geringen Färbeintensitäten zu sein. Da das Gewebeeingangsgut die Etablierung der immunhistochemischen Färbeprotokolle an einzelnen Standorten beeinflusst (z. B. Schnellprozessierung in der Fixierung, Nutzung von hauptsächlich kleinen Biopsaten) und für Ringversuche ältere, große Resektate verwendet werden, ist die schwächer ausgeprägte Färbeintensität der Ringversuchsteilnehmer ggf. hierauf zurückzuführen. Eine stets parallele Analyse eigener Gewebeproben (insbesondere die Gewebeproben der eigenen Etablierung/Validierung) mit den externen Ringversuchsproben im gleichen Färbelauf kann dies absichern.

## Fazit

Die molekularen Nachweise der *BRAF-*V600E-Mutation sind in den pathologischen Instituten sehr gut etabliert und stellen eine äußerst zuverlässige Grundlage für Therapieempfehlungen mittels gezielter Behandlungsoptionen dar. Der Nachweis der *BRAF*-V600E-Mutation mittels Immunhistochemie neigt hingegen zu einer falsch negativen Bewertung von Ringversuchsgeweben und kann ggf. zum Ausschluss von Patienten führen, die aufgrund der immunhistochemisch nicht nachgewiesenen* BRAF*-V600E-Mutation einer entsprechenden Therapie nicht zugeführt werden würden. Daher stellen nach den Ergebnissen des hier beschriebenen *BRAF*-V600E-Ringversuchs die molekularen Methoden die Verfahren der Wahl für den Nachweis von *BRAF*-V600E-Mutationen in pathologischen Instituten als Grundlage für die Therapieentscheidungen dar. Die Immunhistochemie allein kann für die Therapieentscheidung nicht empfohlen werden.
